# Pelvic Floor Myofascial Dysfunction and Lower Urinary Tract Symptoms: A Mechanism-Based Narrative Review and Proposed Neuro-Musculoskeletal Model

**DOI:** 10.3390/medicina62101952

**Published:** 2026-10-09

**Authors:** Jordan Daniel Barber, Christina Biancaniello

**Affiliations:** 1 National Coalition of Independent Scholars (NCIS), Brattleboro, VT 05301, USA; 2 Bioscience Department, Pacific College of Health and Science, New York, NY 10038, USA; christina.bian@pacificcollege.edu

**Keywords:** central nervous system sensitization, lower urinary tract symptoms, myofascial pain syndromes, pelvic floor, trigger points, urinary bladder

## Abstract

*Background and Objectives*: Lower urinary tract symptoms (LUTS), such as urgency, frequency, and pelvic discomfort, are often attributed to bladder-centered mechanisms; nonetheless, several patients exhibit overlapping pelvic floor dysfunctions that existing urologic models do not fully explain. Evidence from the musculoskeletal and pain literature demonstrates that myofascial trigger points within the pelvic floor are prevalent in patients with pelvic pain and urinary symptoms, potentially contributing to symptom generation. In this narrative review, we synthesized the literature on pelvic floor myofascial dysfunction, referred pain patterns, and autonomic regulation of bladder function to develop an integrated neuromusculoskeletal framework. *Materials and Methods*: PubMed and the TRIP Database were searched, with Elicit used for multi-database querying, abstract screening, and assisted data extraction; the final search was performed on 7 April 2026. Eligible publications addressed myofascial trigger point physiology, pelvic floor dysfunction, lower urinary tract function, or mechanisms of central or cross-sensitization and were required to describe or propose a physiological mechanism. Case reports were excluded. Seventeen publications were included in the synthesis. *Results*: The reviewed evidence supports a pathway in which active myofascial trigger points generate sustained nociceptive afferent inputs that converge with bladder afferents in the sacral spinal cord, potentially facilitating cross- and central sensitization, lowering the threshold for bladder sensation, and altering micturition reflex processing. The resulting amplification of afferent signaling provides a mechanistic basis for urgency, frequency, and pelvic pain in the absence of primary bladder pathology. *Conclusions*: This model integrates musculoskeletal and urological perspectives and suggests that, in a subset of patients, LUTS may reflect the central processing of peripheral musculoskeletal input. Incorporating pelvic floor assessment into clinical evaluations may improve diagnostic accuracy and provide more targeted management. Further prospective studies are warranted to validate this integrated pathway and its therapeutic implications.

## 1. Introduction

Lower urinary tract symptoms (LUTS), including urgency, frequency, nocturia, and incontinence, impose a substantial burden on patients and the healthcare system. Overactive bladder syndrome alone affects approximately one in seven adults in the United States, and its prevalence increases with age [1]. Chronic pelvic pain frequently co-occurs with LUTS, with a reported prevalence of 5.7–26.6% in women worldwide; myofascial pelvic floor pain is identified in 14–23% of patients presenting with this condition [2,3]. Nevertheless, pelvic pain and urinary dysfunction have traditionally been managed through separate paradigms, with limited mechanistic integration between urological models focused on the bladder and musculoskeletal models focused on pelvic floor structure and tone.

Conventional approaches emphasize urothelial dysfunction, detrusor overactivity, or bladder outlet obstruction as the primary drivers of symptoms [4,5]. Although well supported, these models may be insufficient to provide a full clinical picture, particularly for patients refractory to bladder-directed therapies [5]. Studies of myofascial pelvic floor dysfunction have demonstrated that trigger points in the pelvic musculature are associated with urgency, frequency, pain, and symptoms that overlap extensively with those attributed to primary bladder pathology [3,6,7,8]. Nonetheless, the mechanisms by which peripheral musculoskeletal pathologies influence nervous system processing and bladder function remain unclear.

This review synthesizes evidence on myofascial trigger points (MTrPs), referred pain patterns, and autonomic bladder regulation to propose an integrated neuromusculoskeletal model tracing a pathway from peripheral musculoskeletal input through spinal afferent processing to alterations in pain perception and urinary function.

Although each component of this pathway is established in its respective literature, these components have not previously been organized into a single explicit sequence connecting pelvic floor myofascial dysfunction with altered micturition. Existing reviews have focused on either myofascial pelvic pain or bladder afferent physiology; by contrast, the present review integrates both domains, delineates the intermediate steps through which somatic input may affect visceral sensory processing, and identifies the points along the pathway that remain empirically unverified.

## 2. Materials and Methods

This narrative, mechanism-based review was designed to synthesize evidence from the musculoskeletal, pain, and urologic literatures and to develop an integrated conceptual model. It was not conducted as a systematic review and does not adhere to PRISMA reporting standards.

Literature was identified through searches of PubMed (National Library of Medicine, Bethesda, MD, USA) and the TRIP Database (Tripdatabase Ltd., Newport, UK), supported by Elicit (Elicit Research, PBC, Oakland, CA, USA) for multi-database querying. Searches were conducted between 15 March 2026 and 7 April 2026. Search terms included myofascial trigger points, pelvic floor, lower urinary tract symptoms, overactive bladder, central sensitization, cross-sensitization, interstitial cystitis, chronic pelvic pain, bladder afferent, and neuromodulation, used individually and in combination.

Publications were eligible if they addressed MTrP physiology, pelvic floor dysfunction, lower urinary tract function, or mechanisms of central or cross-sensitization, and if they described or proposed a physiological mechanism. Preference was given to studies employing objective or standardized assessment. Case reports were excluded, as were reports describing intervention effects without reference to an underlying mechanism; treatment studies were retained where symptom outcomes were interpreted within a proposed mechanistic framework. Animal studies were initially excluded but were subsequently admitted for evidence concerning pelvic organ cross-sensitization, for which corresponding human data were not available.

Elicit was used during three stages of the review process: multi-database literature identification, assisted abstract screening for relevance, and assisted data extraction from full-text articles that had been manually imported after abstract-level selection. All study-selection decisions were made manually by the authors, all extracted data were manually verified, and all included publications were read in full by the authors. Additional studies were identified through review of the reference lists of included articles. In total, 17 publications, consisting of primary studies and mechanistic reviews, were included in the final synthesis.

## 3. Neuroanatomy and Neural Control of the Lower Urinary Tract

The lower urinary tract is innervated by parasympathetic, sympathetic, and somatic pathways, converging from S2 to S4 [5]. Parasympathetic fibers via the pelvic nerve promote detrusor contraction during voiding; moreover, sympathetic fibers from T10 to L2 via the hypogastric nerve promote storage by relaxing the detrusor and contracting the outlet, whereas the somatic pudendal nerve maintains sphincter contraction through the guarding reflex [4,5].

Furthermore, bladder afferents travel primarily in the pelvic nerve with additional contributions from the hypogastric and pudendal nerves [9]. Myelinated Aδ-fibers respond to bladder distension and convey filling sensations at physiologic pressures [5,9]. Unmyelinated C-fibers, which are normally silent, activate in response to noxious or inflammatory stimuli and may develop mechanosensitivity in pathological states, contributing to urgency and bladder overactivity [1,5,9].

Afferent terminals contain substance P and calcitonin gene-related peptides (CGRP), which function as sensory transmitters and mediators of neurogenic inflammation [9]. The suburothelial nerve plexus is particularly dense at the bladder neck and trigone. The urothelium itself acts as a mechanosensor, releasing ATP, nitric oxide, and acetylcholine in response to distension, thereby modulating afferent nerve activity and micturition reflex initiation [4,9]. Bladder sensory processing is dynamically modulated by local chemical signaling rather than operating as a fixed system.

The bladder afferent synapses in the sacral spinal cord, where ascending projections reach the pontine micturition center (PMC), coordinate the storage-voiding transitions [5]. The sacral dorsal horn is a convergence site for afferent input from the bladder, bowel, and pelvic floor musculature [1,10], providing a neuroanatomical substrate for the interaction between musculoskeletal input and bladder sensory processing.

## 4. Myofascial Trigger Points in the Pelvic Floor

### 4.1. Prevalence and Clinical Significance

MTrPs are hyperirritable foci within the taut bands of the skeletal muscles that produce local and referred pain [8,11]. In the MAPP Research Network, 81% of the participants with urologic chronic pelvic pain syndrome (UCPPS) exhibited pelvic floor tenderness compared with 9% of the controls [12]. Similarly, 78% of patients with interstitial cystitis have at least one identifiable trigger point [7], and MTrPs may be present in up to 85% of urologic pelvic pain patients [8].

These findings extend beyond the pain. In a cohort of patients with pelvic floor myofascial syndrome, 67.6%, 60.3%, and 57.4% reported nocturia, frequency, and urgency, respectively [3]. Einig et al. demonstrated correlations between trigger point pain and overall severity of LUTS [2], whereas MAPP Research Network data associated more widespread pelvic floor tenderness with greater symptom burden and reduced quality of life [12].

Pelvic floor tenderness and discretely identified active MTrPs are related but separate constructs. Tenderness detected during palpation reflects broader pelvic floor involvement and does not necessarily indicate the presence of a discrete taut band with a characteristic referral pattern. Several studies cited in this review assessed tenderness rather than formally diagnosed MTrPs; this distinction is therefore maintained in the descriptions that follow.

### 4.2. Involved Musculature and Biochemistry

The levator ani complex is most commonly affected, with high tenderness rates in patients with chronic pelvic pain [13]. The obturator internus is also frequently involved and has been specifically associated with urinary urgency [2]. Beyond the pelvic floor muscles, MTrPs in adjacent structures such as the rectus abdominis and external obliques have been found to reproduce suprapubic, groin, and genital pain, demonstrating that the relevant nociceptive input may originate outside the pelvic floor itself [14]. These findings support a broader view of pelvic dysfunction involving an integrated musculoskeletal network, rather than isolated muscles.

Active MTrPs exhibit elevated levels of bradykinin, substance P, CGRP, tumor necrosis factor-α, and serotonin, along with reduced pH [8]. These same mediators are implicated in bladder C-fiber activation and neurogenic inflammation [5,9]. Elevated mediators have also been observed in remote, uninvolved muscles, suggesting biochemical effects beyond local tissue [8].

### 4.3. Association with Urinary Symptoms

Pelvic floor tenderness was correlated with symptom severity in patients with interstitial cystitis [7]. In some patients, urinary frequency increases even when no primary bladder pathology is identified, reflecting underlying myofascial dysfunction [6]. Manual therapy targeting pelvic floor hypertonicity has produced moderate to marked improvement in urgency–frequency syndromes [15], and combined myofascial release with relaxation training has yielded significant improvements in refractory chronic pelvic pain [16]. These findings are consistent with pelvic floor myofascial dysfunction acting as a source of afferent input capable of influencing nervous system processing.

### 4.4. Referred Pain and Symptom Misattribution

MTrPs characteristically generate pain at sites that are distant from their origin. In the pelvis, referral patterns follow the predictable trajectories described by Travell and Simons and have been confirmed by subsequent clinical studies [11]. The territories to which pelvic floor MTrPs refer pain overlap extensively with regions where patients with urological, gynecological, and colorectal conditions report symptoms; therefore, musculoskeletal pathology may mimic primary organ disease.

Anderson et al. demonstrated that puborectalis and pubococcygeus MTrPs reproduced penile pain in over 75% of cases. Conversely, external oblique palpation elicited suprapubic, testicular, and groin pain in at least 80% of cases [14]. Moreover, Weiss [15] reported that the levator ani pressure refers to pain in the suprapubic region, perineum, rectum, glans penis, and labia. Finally, obturator internus tenderness has been associated with vulvar and perineal referrals and urinary urgency [2,13].

The resulting symptoms may be indistinguishable from those of bladder pathology [6,13]. Ackerman and Wise-Fuchs [6] have named pelvic floor myofascial dysfunction a “great pretender,” producing frequency, urgency, and sensations of incomplete emptying routinely attributed to bladder disorders. Bassaly et al. [7] observed that pain elicited during the examination of interstitial cystitis patients often originates from structures other than the bladder, and Itza et al. [11] noted that multiple distinct diagnoses, including chronic prostatitis, interstitial cystitis, levator ani syndrome, and vulvodynia, share myofascial pain as a common component.

When pelvic floor examination is omitted, a musculoskeletal source of symptoms may go undetected, and treatment directed exclusively at the bladder may yield suboptimal results. The finding that myofascia-directed therapies improve urinary symptoms in patients with overlapping presentations [15,16] is consistent with a musculoskeletal contribution to the symptom burden.

Referred pain also supports the concept of shared spinal processing; nociceptive input from the pelvic floor muscles converges on dorsal horn neurons that also receive bladder afferents from S2 to S4 [1,5,9,10]. Reproducible referral patterns support the view that musculoskeletal inputs access the same spinal circuits that process bladder information.

### 4.5. Autonomic Regulation and Afferent Vulnerability

Bladder function occurs in two mutually exclusive modes, storage and elimination, governed by reciprocal autonomic activity coordinated through spinal and supraspinal circuits [5]. During storage, sympathetic outflow maintains detrusor relaxation and outlet contraction, whereas parasympathetic input is suppressed. Pudendal nerve activity contracts the external sphincter through the guarding reflex [4,5]. When intravesical volume reaches the micturition threshold, ascending afferent signals activate the PMC, which coordinates parasympathetic-driven detrusor contraction with inhibition of sympathetic and somatic outflow [5]. Any change in afferent signaling reaching the sacral cord may shift this threshold, producing urgency, increased frequency, or involuntary detrusor contractions [1,5,9].

During normal filling, continuous low-level afferent activity (“afferent noise”) from bladder mechanoreceptors is transmitted to the sacral cord via Aδ-fibers [1]. Only a fraction reaches conscious awareness; most contributes to subconscious reflexes coordinating sphincter function and detrusor quiescence. This input is filtered by the central nervous system, with nonessential signals suppressed while threshold events that initiate voiding are monitored [1].

Two perturbations may disrupt this filtering. First, bladder-derived input may be increased because of urothelial dysfunction, detrusor micromotion, or altered receptor sensitivity [1,4]. Second, central processing may be altered so that input that is normally subthreshold is facilitated to suprathreshold levels. Reynolds et al. proposed that this central amplification of afferent signaling may underlie bladder hypersensitivity in idiopathic overactive bladders, in which urgency occurs at reduced volumes without a clear structural pathology [1].

Autonomic regulation of the bladder exhibits substantial plasticity in response to sustained changes in afferent input. Yoshimura et al. described how neurotrophic factors, such as nerve growth factors, enhance afferent neuron excitability and modify spinal synaptic transmission, thereby contributing to bladder overactivity [5]. Although this has been demonstrated primarily in bladder-centered conditions, the principle is not restricted to bladder-derived inputs. If sustained nociceptive signaling from pelvic floor MTrPs reaches shared sacral circuits, similar neuroplastic mechanisms may be engaged, contributing to altered autonomic regulation independent of intrinsic bladder disease.

Ochodnicky et al. [4] emphasized that autonomic bladder control extends beyond classical efferent regulation. Neurotransmitter receptors on the urothelium, interstitial cells, and afferent terminals form a distributed sensory network that modulates afferent output during filling, underscoring the susceptibility of these pathways to modulation by extrinsic nociceptive inputs.

## 5. Mechanisms Linking Musculoskeletal Input to Bladder Dysfunction

The preceding sections established three foundational observations: pelvic floor MTrPs are a prevalent source of nociceptive afferent input; this input arises from anatomical territories overlapping with bladder-attributed symptoms; and bladder autonomic regulation is fundamentally afferent-dependent and susceptible to perturbations. This section integrates these observations into a mechanistic sequence, tracing how somatic musculoskeletal inputs from the pelvic floor may be processed through the nervous system to produce changes in pain perception and urinary function.

### 5.1. Peripheral Nociceptive Input

Sustained nociceptive afferent activity is generated by active MTrPs through elevated concentrations of bradykinin, substance P, CGRP, and proinflammatory cytokines, with reduced tissue pH [8]. This input is transmitted to the sacral cord via pudendal and pelvic nerve branches [5,13]. Two features are mechanistically significant: the input is sustained, providing the repetitive barrage necessary to induce central changes [1,8]; and the neuropeptides released (substance P and CGRP) are the same mediators involved in bladder C-fiber signaling, where they function as sensory transmitters and mediators of neurogenic inflammation [8,9]. This molecular overlap suggests that pelvic floor-derived nociceptive input engages the same signaling systems that modulate bladder afferent processing.

### 5.2. Dorsal Horn Convergence

At the sacral dorsal horn, pelvic floor afferents converge with bladder and bowel afferents on shared second-order neurons. The cross-sensitization theory proposes that sustained nociceptive input from one structure increases the excitability of shared neurons, leading to exaggerated responses to input from other structures [10]. In animal studies, dichotomizing sensory fibers and single dorsal root ganglion neurons have been identified, with axons branching to innervate multiple pelvic structures, thereby providing an additional peripheral route for cross-organ influence [10]. Altered afferent processing within the dorsal root ganglia, including changes in electrical coupling and chemical signaling between adjacent cell bodies, has been proposed as a peripheral mechanism of cross-sensitization [10].

A practical implication is that nociceptive input from pelvic floor MTrPs does not remain confined to somatic pathways. Instead, it reaches spinal neurons that process bladder signals, creating a route by which musculoskeletal inputs may influence visceral sensory processing.

### 5.3. Central Sensitization

Sustained peripheral nociceptive input may induce central sensitization, defined as increased responsiveness of central nociceptive neurons to normal or subthreshold afferent input [1]. Reynolds et al. [1] described this process as driven by repetitive activation of C-fiber afferents synapsing in the dorsal horn, resulting in heterosynaptic potentiation: both nociceptive C-fiber signals are amplified, and afferent signals from low-threshold Aδ and Aβ mechanoreceptors, which normally generate nonpainful sensations, are facilitated to suprathreshold levels. Sensitized spinal neurons exhibit reduced firing thresholds, expanded receptive fields, and ongoing, stimulus-independent activity [1].

At the molecular level, NMDA receptor activation and signaling by substance P, CGRP, and nerve growth factor contribute to increased neuronal excitability and synaptic transmission in the dorsal horn [1]. These mediators also overlap with those involved in bladder afferent signaling, supporting a shared biochemical basis for cross-system influence. Because neural circuits converge at the spinal level, the hypersensitivity associated with central sensitization can extend to areas remote from the conditioning stimulus, potentially contributing to symptom spread [1].

Evidence supports the relevance of central sensitization to bladder dysfunction. In a systematic review, Knox et al. found consistent evidence of central sensitization in all 15 studies of bladder pain syndrome, and greater sensitization was associated with more severe symptoms and more comorbidities [17]. Similarly, MAPP Research Network data demonstrated that patients with more widespread pelvic floor tenderness exhibited greater symptom severity and patterns consistent with centralized pain processing [12]. These findings support the hypothesis that peripheral musculoskeletal inputs contribute to the afferent drive that sustains central sensitization.

### 5.4. Cross-Sensitization Between the Pelvic Floor and the Bladder

Cross-sensitization represents a specific application of central sensitization to interactions among pelvic structures. Experimental studies have demonstrated that pathology in one pelvic organ can alter the sensitivity of afferents innervating adjacent organs via shared spinal pathways [10]. Reynolds et al. [1] extended this concept to clinical observations, suggesting that central sensitization initiated in one organ system may spread to related neural circuits and produce overlapping symptom patterns.

Although the cross-sensitization literature has focused predominantly on viscerovisceral interactions, similar neuroanatomical principles also apply to somatovisceral interactions between the pelvic floor musculature and the bladder. Somatic afferents from the pelvic muscles and visceral afferents from the bladder converge within the sacral spinal segments [5,9,10]. Sustained nociceptive input arising from pelvic floor MTrPs may amplify afferent processing of bladder signals and lower the threshold at which filling is perceived as urgent. This somatovisceral cross-sensitization provides a plausible mechanism through which pelvic floor dysfunction may produce urinary symptoms, even without identifiable bladder dysfunction.

### 5.5. Descending Modulation

Central sensitization is further influenced by descending modulation from supraspinal centers, which can facilitate or inhibit afferent processing. In states of sensitization, descending facilitation may contribute to the amplification and spread of symptoms beyond the initial territory of dysfunction [1]. Because descending projections are typically more diffuse and bilateral than spinal projections, they may explain the clinical observation that pelvic pain and urinary symptoms extend well beyond the territory of the originally involved muscles.

Clinical observations suggest that pelvic floor myofascial dysfunction may precede, or contribute to, more complex pelvic pain syndromes, potentially through sustained afferent input driving progressive neural sensitization [15]. Although unconfirmed by prospective studies, this concept aligns with evidence that greater pelvic floor tenderness is associated with more centralized pain phenotypes [1,12]. The key evidence supporting each domain of this pathway is summarized in Table 1.

### 5.6. Proposed Neuromusculoskeletal Model

**Figure 1:**
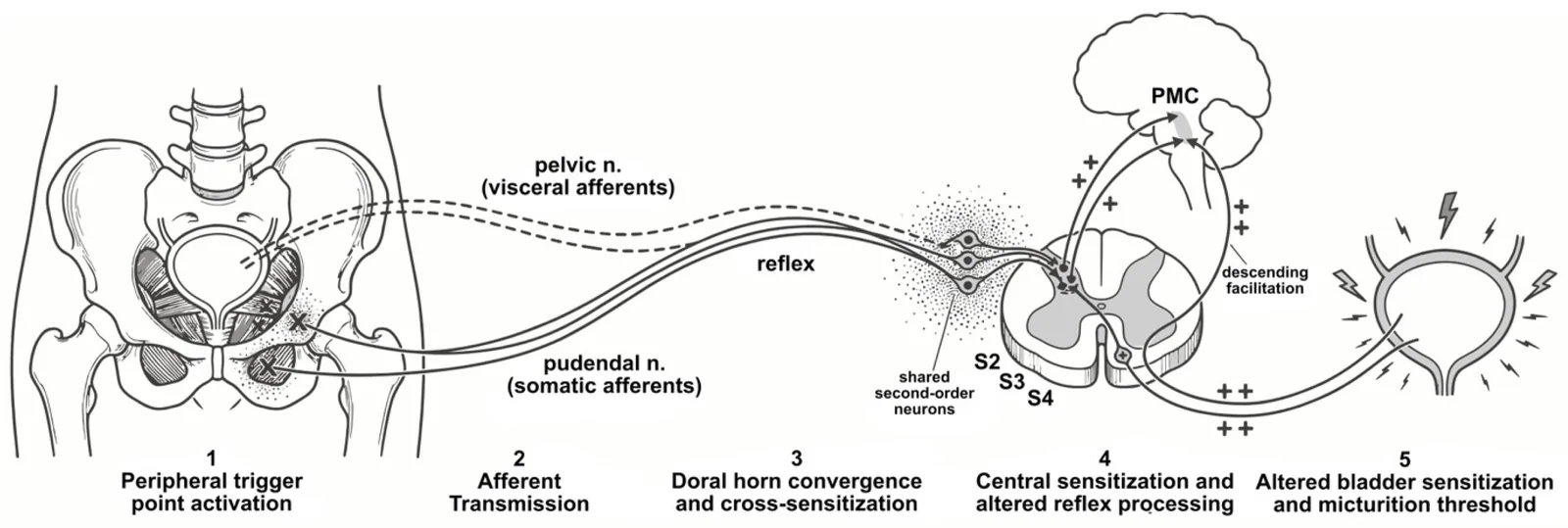
Five-stage integrated model overview.

We propose an integrated five-stage model (Figure 1) in which pelvic floor myofascial dysfunction contributes to pain and urinary symptoms through a specified physiological pathway. This model does not replace bladder-centric or musculoskeletal frameworks; instead, it links them. Each component is supported by existing evidence, but the complete pathway remains to be tested prospectively.

#### 5.6.1. Stage 1: Peripheral Trigger Point Activation

Active MTrPs in the pelvic floor and associated musculature, characterized by sustained contraction and an inflammatory biochemical environment [8,12], may activate and sensitize peripheral nociceptors, generating persistent afferent signaling.

#### 5.6.2. Stage 2: Afferent Transmission to the Sacral Spinal Cord

Nociceptive signals travel via the pudendal and pelvic nerves to S2–S4, where bladder afferents are also processed [5,9,10]. These signals use neuropeptide systems shared with bladder C-fiber pathways, including substance P and CGRP, which participate in common mechanisms of afferent signaling.

#### 5.6.3. Stage 3: Dorsal Horn Convergence and Cross-Sensitization

Within the sacral dorsal horn, somatic afferents from the pelvic muscles converge with visceral afferents from the bladder on shared second-order neurons [1,10]. Sustained nociceptive input may increase neuronal excitability, producing cross-sensitization. Normal bladder input may then be amplified and perceived as heightened urgency, pressure, or discomfort.

#### 5.6.4. Stage 4: Central Sensitization and Altered Reflex Processing

Persistent convergent inputs may induce central sensitization in spinal and supraspinal circuits. Sensitized neurons exhibit reduced activation thresholds and increased responsiveness [1]. Clinically, this may lower the threshold for bladder sensation and for activation of the micturition reflex, potentially contributing to urgency, frequency, and in some cases involuntary detrusor activity. Descending modulatory pathways may further reinforce this sensitized state.

#### 5.6.5. Stage 5: Clinical Expression

The clinical output is a constellation of symptoms, including pelvic pain and lower urinary tract dysfunction. In this model, these symptoms arise from integrated processing of convergent somatic and visceral inputs within a sensitized nervous system. This framework is consistent with the observed prevalence of MTrPs in bladder-related syndromes, overlapping referral patterns, and improvements following myofascial interventions [7,8,12,14,15,16].

### 5.7. Properties of the Model

The model is bidirectional (bladder pathology may also drive pelvic floor dysfunction), graded (severity varies with the intensity and duration of peripheral input) [12], and potentially progressive. It is clinically actionable, and the peripheral MTrP represents a modifiable input to a central process. Evidence that myofascial interventions improve both pain and urinary outcomes [15,16] is consistent with this framework. Nevertheless, prospective studies testing the full mechanistic chain are required.

## 6. Discussion

A purely urological model cannot account for the high prevalence of pelvic floor MTrPs in bladder pain cohorts [7,8,12], reproduction of bladder symptoms by pelvic floor palpation [14,15], or improvement of urinary symptoms following musculoskeletal interventions [15,16]. Conversely, a purely musculoskeletal model cannot explain the autonomic reflex changes, central sensitization, or neuroplastic alterations observed during afferent processing [1,5,17]. These findings support an integrated framework in which peripheral musculoskeletal inputs and visceral afferent processing interact via shared spinal circuits.

This model supports incorporating pelvic floor musculoskeletal assessment into the standard evaluation of patients with LUTS, particularly those refractory to conventional bladder-directed therapy. Given the high prevalence of pelvic floor tenderness in UCPPS populations [12] and the fact that detailed pelvic floor examinations are not routinely taught in urology or gynecology training [13], a musculoskeletal source of symptoms may go unrecognized when this evaluation is omitted. Patients may then undergo prolonged, ineffective, bladder-focused treatment, at least in part, for peripheral musculoskeletal conditions [6]. Einig et al. demonstrated that a standardized trigger-point examination correlates with severity of LUTS, supporting its clinical utility [2]. This recommendation rests most securely on evidence from pain-predominant cohorts; its applicability to patients with isolated storage symptoms remains to be established.

In this model, urgency may reflect central amplification of normal filling signals within a sensitized spinal cord. Urinary frequency may result from a lowered micturition threshold driven by convergent musculoskeletal input. Suprapubic discomfort may represent referred pain from pelvic floor MTrPs [6,13,15]. If pelvic floor MTrPs serve as peripheral drivers of centrally mediated urinary symptoms, interventions that reduce trigger-point activity may address both local pain and the afferent input sustaining central sensitization. Manual therapy has produced moderate to marked improvements in urgency–frequency syndromes [15], and combined myofascial release with relaxation training has yielded clinically significant improvements in refractory chronic pelvic pain [16].

Randomized evidence also bears on this question. In a multicenter randomized controlled trial of women with interstitial cystitis/bladder pain syndrome and demonstrable pelvic floor tenderness, myofascial physical therapy produced moderate or marked improvement in 59% of participants compared with 26% receiving global therapeutic massage, and a parallel multicenter trial in patients with chronic prostatitis/chronic pelvic pain syndrome or interstitial cystitis/bladder pain syndrome reported a global response rate of 57% with myofascial physical therapy versus 21% with global therapeutic massage [7,8,12]. Manual physical therapy directed at high-tone pelvic floor dysfunction is correspondingly recommended as a second-line intervention in current guidance for interstitial cystitis/bladder pain syndrome [8]. Notably, improvement in these trials was observed on global response measures rather than on discrete urinary or pain parameters, and participants were not stratified by the extent or severity of pelvic floor tenderness [12]. Prospective controlled trials therefore remain warranted to determine whether the proposed mechanistic pathway accounts for the observed treatment effects.

### Limitations

The neuromusculoskeletal pathway has not been tested as an integrated sequence within a single prospective study. Although evidence supports each component, studies supporting individual stages involve different populations, methodologies, and outcome measures. Thus, the inference that these stages constitute a connected physiological pathway relies on cross-domain synthesis rather than direct experimental validation.

The evidence linking pelvic floor MTrPs to urinary symptoms is predominantly observational and cross-sectional [2,3,7,12]. These designs can establish associations but do not determine causation or directionality. It remains unclear whether MTrPs are a primary driver of urinary symptoms, a secondary consequence of bladder pathology, or a co-occurring phenomenon arising from shared underlying mechanisms.

The evidence synthesized in this review comes from heterogeneous populations, including interstitial cystitis/bladder pain syndrome, urologic chronic pelvic pain syndrome, chronic prostatitis/chronic pelvic pain syndrome, and myofascial pelvic pain. Although these populations overlap, they are not equivalent, and each is pain-predominant. Evidence directly addressing patients with storage LUTS without pelvic pain remains comparatively limited; the prevalence, referral, and treatment findings discussed here are drawn almost entirely from cohorts defined by pain. Extending the proposed model to non-painful storage LUTS is therefore an inference based on mechanistically related but clinically distinct populations and requires direct validation in patients who do not have a pain phenotype.

Cross-sensitization evidence has largely been derived from animal models of viscerovisceral interactions, particularly bowel-to-bladder sensitization [10]. The applicability of these findings to somatovisceral interactions between the pelvic floor musculature and the bladder in humans has not been directly investigated. Diagnostic criteria for pelvic floor MTrPs have not yet been standardized. Examination methods differ across studies, and the diagnosis has not been confirmed by validated laboratory or imaging modalities [2,13]. This heterogeneity creates uncertainty regarding both prevalence and clinical significance. A related limitation is that several of the largest datasets cited here, including those from the MAPP Research Network, evaluated pelvic floor tenderness rather than formally diagnosed active MTrPs. Tenderness represents a broader and less specific finding, and the extent to which it reflects the trigger-point pathology central to the proposed model remains unestablished. This limitation also applies to the randomized treatment evidence, in which participants were enrolled on the basis of pelvic floor tenderness rather than formally diagnosed MTrPs [7,8,12].

Evidence of central sensitization in an overactive bladder without pain remains indirect. Quantitative sensory testing has not been systematically applied in this population [1], and a systematic review by Knox et al. focused primarily on bladder pain syndrome rather than non-painful urinary dysfunction [17]. Although randomized trials support myofascial physical therapy in pelvic pain populations [7,8,12], the studies bearing most directly on the proposed mechanism [15,16] are limited by small sample sizes, lack of randomization, and absence of blinding. Moreover, no trial has yet tested whether symptom improvement following myofascial intervention is mediated by the afferent and central mechanisms proposed here; demonstrating that a treatment works does not establish why it works. Prospective, mechanistically informed trials integrating musculoskeletal assessments, neurophysiological measures, and urodynamic outcomes are needed to test this framework.

## 7. Conclusions

This review proposes an integrated neuromusculoskeletal model of pelvic floor-mediated LUTS, outlining a pathway from active MTrPs to sustained afferent signaling, spinal convergence to central sensitization, and altered bladder function. Each component is supported by published evidence; however, the complete sequence has not yet been tested as an integrated chain.

A principal implication is that, in a subset of patients, LUTS may reflect central processing of peripheral musculoskeletal input rather than solely intrinsic bladder pathology. This study suggests that a comprehensive evaluation should include a pelvic floor assessment. The contribution of this review is integrative rather than empirical. It consolidates findings that currently reside in separate literatures into a single pathway specified in sufficient detail to be tested, and identifies the specific junctions at which that pathway lacks direct evidence. This framework also provides a basis for prospective studies integrating musculoskeletal, neurophysiological, and urodynamic outcomes.

## Figures and Tables

**Table 1:** Key evidence by domain.

Domain	Key Finding	Representative Source
Trigger Points	81% of UCPPS participants had pelvic floor tenderness vs. 9% of controls; widespread tenderness was associated with worse symptoms and a centralized pain phenotype	Gupta et al. (2022) [12]
Trigger Points	78% of IC patients had at least one trigger point; 68% had multiple sites	Bassaly et al., 2010 [7]
Trigger Points	Active trigger points contain elevated substance P, CGRP, bradykinin, TNF-α, serotonin, and norepinephrine with reduced pH; elevations also in remote uninvolved muscle	Moldwin and Fariello (2013) [8]
Trigger Points	Pelvic floor myofascial syndrome was associated with nocturia (67.6%), frequency (60.3%), urgency (57.4%)	Leyva Vazquez et al. (2024) [3]
Trigger Points	5 of 7 pain sites reproduced ≥50% of the time by palpation of designated trigger points	Anderson et al. (2009) [14]
Trigger Points	Manual therapy produced moderate to marked improvement in 83% of urgency–frequency patients and 70% of IC patients	Weiss (2001) [15]
Trigger Points	Combined myofascial release and relaxation training yielded improvement in 72% of men with CP/CPPS refractory to conventional therapy	Anderson et al. (2005) [16]
Trigger Points	Trigger point pain in the pubococcygeus, iliococcygeus, and obturator internus correlated with LUTS severity and high pelvic floor tone	Einig et al. (2024) [2]
Trigger Points	MPFD was identified as a hidden contributor to LUTS without primary bladder pathology (myofascial urinary frequency syndrome)	Ackerman and Wise-Fuchs (2026) [6]
Autonomic/Afferent Signaling	Bladder Aδ-fibers convey a filling sensation; C-fibers are normally silent but become mechanosensitive in pathological states; the urothelium releases ATP, NO, and ACh as mechanosensory mediators	Andersson (2002) [9]
Autonomic/Afferent Signaling	Storage-voiding switch governed by reciprocal autonomic activity; NGF-mediated neuroplasticity alters afferent excitability and spinal reflex processing	Yoshimura et al. (2014) [5]
Autonomic/Afferent Signaling	Neurotransmitter receptors on urothelium, interstitial cells, and afferent nerves form a distributed sensory network; non-neuronal ACh released during storage increases with age	Ochodnicky et al. (2013) [4]
Sensitization	Central sensitization produces heterosynaptic potentiation: repetitive C-fiber input amplifies subthreshold Aδ/Aβ signals; proposed mechanism for bladder hypersensitivity in idiopathic OAB	Reynolds et al. (2016) [1]
Sensitization	Cross-sensitization: afferents from different pelvic structures converge onto shared dorsal horn neurons; disease in one organ increases excitability of neurons processing input from adjacent organs	Panicker et al., 2019 [10]
Sensitization	Systematic review found evidence of central sensitization in all 15 BPS studies; greater sensitization correlated with symptom severity and comorbid chronic pain	Knox et al. (2024) [17]

Abbreviations: UCPPS, urologic chronic pelvic pain syndrome; IC, interstitial cystitis; CGRP, calcitonin gene-related peptide; TNF-α, tumor necrosis factor-α; LUTS, lower urinary tract symptoms; CP/CPPS, chronic prostatitis/chronic pelvic pain syndrome; NGF, nerve growth factor; OAB, overactive bladder; MPFD, myofascial pelvic floor dysfunction; BPS, bladder pain syndrome; ACh, acetylcholine.

## Data Availability

No new data were generated or analyzed to support this research. All the information presented was derived from previously published studies cited in the manuscript.

## Abbreviations

The following abbreviations are used in this manuscript:

ATP	Adenosine triphosphate
CGRP	Calcitonin gene-related peptide
CP/CPPS	Chronic prostatitis/chronic pelvic pain syndrome
DRG	Dorsal root ganglion
IC/BPS	Interstitial cystitis/bladder pain syndrome
LUTS	Lower urinary tract symptoms
MAPP	Multidisciplinary Approach to the Study of Chronic Pelvic Pain
MPFD	Myofascial pelvic floor dysfunction
MTrPs	Myofascial trigger points
NGF	Nerve growth factor
NMDA	N-methyl-D-aspartate
OAB	Overactive bladder
PMC	Pontine micturition center
TNF-α	Tumor necrosis factor-α
TRIP	Turning Research Into Practice
UCPPS	Urologic chronic pelvic pain syndrome
